# A lipoprotein lipase–GPI-anchored high-density lipoprotein–binding protein 1 fusion lowers triglycerides in mice: Implications for managing familial chylomicronemia syndrome

**DOI:** 10.1074/jbc.RA119.011079

**Published:** 2019-10-23

**Authors:** Amitabh V. Nimonkar, Stephen Weldon, Kevin Godbout, Darrell Panza, Susan Hanrahan, Rose Cubbon, Fangmin Xu, John W. Trauger, Jiaping Gao, Andrei Voznesensky

**Affiliations:** ‡ Cardiovascular and Metabolic Disease Area, Novartis Institutes for Biomedical Research, Cambridge, Massachusetts 02139; § Novartis Biologics Center, Novartis Institutes for Biomedical Research, Cambridge, Massachusetts 02139; ¶ Protein Analytics, Novartis Institutes for Biomedical Research, Cambridge, Massachusetts 02139

**Keywords:** cell metabolism, lipid metabolism, lipoprotein, pharmacology, protein engineering, lipase, triglyceride, familial chylomicronemia syndrome, FCS, GPIHBP1, lipoprotein lipase, pancreatitis

## Abstract

Lipoprotein lipase (LPL) is central to triglyceride metabolism. Severely compromised LPL activity causes familial chylomicronemia syndrome (FCS), which is associated with very high plasma triglyceride levels and increased risk of life-threatening pancreatitis. Currently, no approved pharmacological intervention can acutely lower plasma triglycerides in FCS. Low yield, high aggregation, and poor stability of recombinant LPL have thus far prevented development of enzyme replacement therapy. Recently, we showed that LPL monomers form 1:1 complexes with the LPL transporter glycosylphosphatidylinositol-anchored high-density lipoprotein–binding protein 1 (GPIHBP1) and solved the structure of the complex. In the present work, we further characterized the monomeric LPL/GPIHBP1 complex and its derivative, the LPL–GPIHBP1 fusion protein, with the goal of contributing to the development of an LPL enzyme replacement therapy. Fusion of LPL to GPIHBP1 increased yields of recombinant LPL, prevented LPL aggregation, stabilized LPL against spontaneous inactivation, and made it resistant to inactivation by the LPL antagonists angiopoietin-like protein 3 (ANGPTL3) or ANGPTL4. The high stability of the fusion protein enabled us to identify LPL amino acids that interact with ANGPTL4. Additionally, the LPL–GPIHBP1 fusion protein exhibited high enzyme activity in *in vitro* assays. Importantly, both intravenous and subcutaneous administrations of the fusion protein lowered triglycerides in several mouse strains without causing adverse effects. These results indicate that the LPL–GPIHBP1 fusion protein has potential for use as a therapeutic for managing FCS.

## Introduction

Lipoprotein lipase (LPL)^2^ is a triglyceride lipase secreted primarily by adipocytes, skeletal muscle cells, and cardiomyocytes. LPL folding is mediated by the chaperone lipase maturation factor 1 (LMF1). LPL is secreted into the subendothelial space and then translocated across endothelial cells to the lumen of capillaries by glycosylphosphatidylinositol-anchored high-density lipoprotein–binding protein 1 (GPIHBP1). After translocation, LPL is tethered to the surface of capillary endothelial cells by heparan sulfate proteoglycans or GPIHBP1. Tethered LPL catalyzes the hydrolysis of triglycerides (TG) carried in very-low-density lipoproteins (VLDL) and chylomicrons (CM) (1). Free fatty acids liberated by LPL are used as a source of energy by the heart and muscle or are stored in the form of TG by adipose tissue. LPL is a tightly controlled enzyme that is stimulated by apolipoprotein C2 and inhibited by ANGPTL3, ANGPTL4, and ANGPTL8 (2). Loss-of-function mutations in LPL, GPIHBP1, or LMF1 result in LPL deficiency, which causes accumulation of TG-rich CM in the blood.

Familial chylomicronemia syndrome (FCS) is a rare genetic disorder caused by LPL deficiency. Patients with FCS exhibit severe hypertriglyceridemia (TG >1,000 mg/dl *versus* normal TG <150 mg/dl). They suffer from nausea, vomiting, eruptive xanthomas, lipemia retinalis, hepatosplenomegaly, and experience recurrent episodes of mild to incapacitating abdominal pain. The most dangerous manifestation of FCS is hypertriglyceridemic pancreatitis (HTAP). HTAP attacks occur in 25–60% of patients with FCS (3–5). The risk of HTAP increases progressively as TG levels increase (6) and rises sharply when triglyceride levels reach 20 mmol/liter (∼1,800 mg/dl) (7). The overall mortality rate for acute pancreatitis is 5–6% but increases to 30% in subgroups of markedly hypertriglyceridemic patients. These subjects experience pancreatic necrosis following an infected pancreatic abscess or persistent multiple organ failure (8).

No specific approved pharmacological intervention has been demonstrated to improve the clinical course of HTAP. Therapeutic options for acutely lowering TG to a safe level (<1000 mg/dl) for the treatment of HTAP are limited to switching patients to parenteral hypocaloric nutrition combined with supportive care. Plasmapheresis is used if the equipment is available (9–11). Prevention of HTAP is also difficult, and patients who have FCS have few options to maintain plasma TG in the safe range and stave off attacks of abdominal pain and pancreatitis. Patients with FCS must restrict their dietary fat to less than 20 g/day or 15% of total energy intake for their entire lives. Approximately 80% of patients with FCS rate this adherence as “very difficult” (12).

For decades, the enzymatically active form of LPL was believed to be a head-to-tail homodimer that dissociates into inactive LPL monomers. On the contrary, we and others recently showed that LPL is active as a monomer. We observed that LPL forms a 1:1 complex with GPIHBP1, showed that the complex is enzymatically active, and solved the crystal structure of this complex (13). In the present work, we provide further evidence that monomeric LPL/GPIHBP1 complex is functionally active and stable.

Capitalizing on the high stability and activity of this monomeric LPL/GPIHBP1 complex, we fused LPL to GPIHBP1. Linking LPL into a covalent complex with GPIHBP1 further increased its resistance to inactivation by the LPL inhibitors ANGPTL3 and ANGPTL4. The stability of the fusion protein allowed us to map the site of interaction of LPL with ANGPTL4 by hydrogen–deuterium exchange. LPL–GPIHBP1 fusion protein showed high enzymatic activity in *in vitro* assays using surrogate substrates as well as the natural LPL substrates VLDL and CM. In several strains of mice, intravenous (i.v.) or subcutaneous (s.c.) administration of the LPL–GPIHBP1 fusion protein lowered plasma TG without adverse effects. LPL–GPIHBP1 fusion protein has properties that favor its development as an agent for the treatment and prevention of hyperlipidemic pancreatitis and/or abdominal pain attacks.

## Results

### GPIHBP1 stabilizes LPL, prevents its aggregation, and increases lipase activity

**Figure 1.**
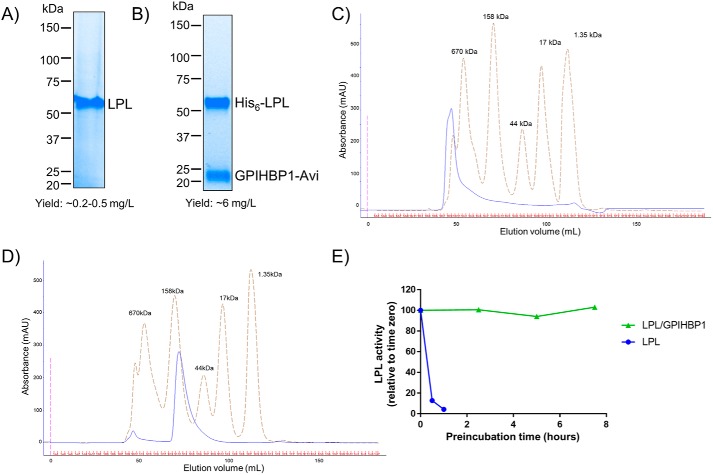
**GPIHBP1 enhances purity, yield, folding, activity, and stability of LPL.** *A*, gel image showing purified human LPL resolved under reducing conditions using 4–20% SDS-PAGE. Untagged LPL was purified using heparin and size-exclusion chromatography. *B*, gel image showing purified human LPL/GPIHBP1 complex resolved under reducing conditions using 4–20% SDS-PAGE. His_6_-LPL and GPIHBP1-Avi were coexpressed in the presence of LMF1. The complex was purified using Ni-affinity and size-exclusion chromatography. *C* and *D*, gel-filtration analysis showing resolution of LPL and LPL/GPIHBP1 complex using a S200 Superdex column, respectively. The chromatogram of molecular weight standards is superimposed on both panels. *E*, the LPL/GPIHBP1 complex is more enzymatically stable than LPL alone. The ability of 10 nm LPL alone (*blue*) and LPL/GPIHBP1 complex (*green*) to hydrolyze VLDL is compared as a function of the incubation time in PBS at room temperature. Enzyme activities are reported relative to time 0. *mAU*, milli-absorbance units.

We initially attempted to express and purify LPL protein alone. We synthesized a variety of LPL constructs that were either untagged or had N- or C-terminal tags (Fig. S1), expressed them in mammalian cells, and purified them using heparin chromatography or Ni-affinity chromatography. We found that the purified proteins were active but were obtained with low yield and were highly aggregated (Fig. 1, *A* and *C*). Coexpression of chaperones is one of the approaches used to optimize the expression of recombinant proteins (14). Cotransfection with the LPL chaperone protein, LMF1, has been reported to improve the yield of recombinant LPL (15). We, on the contrary, observed that cotransfection with LMF1 did not substantially improve LPL yield, and the purified protein was still highly aggregated (Fig. S2).

**Figure 2.**
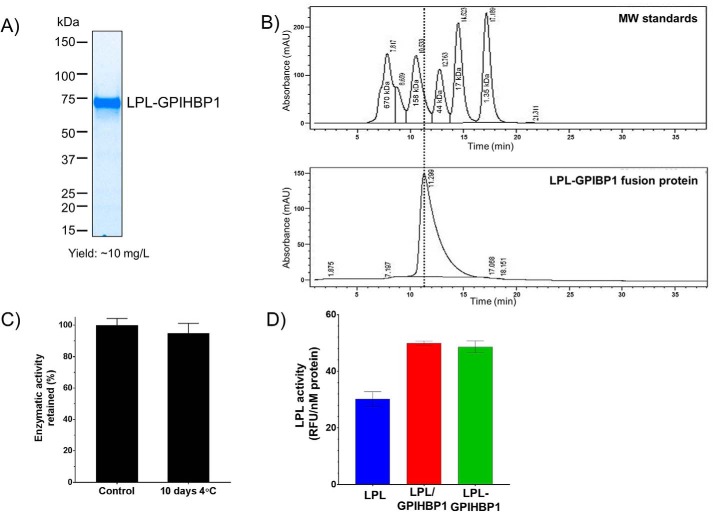
**LPL–GPIHBP1 fusion protein is homogenous and stable and possesses high specific activity.** *A*, gel image showing purified LPL–GPIHBP1 fusion protein resolved under reducing conditions using 4–20% SDS-PAGE. The fusion protein with a C-terminal FHA tag was purified using Ni-affinity and size-exclusion chromatography. *B*, analytical size-exclusion chromatography showing that the fusion protein is free of aggregates and resolves as a homogenous species with approximate molecular weight of ∼75 kDa. *C*, LPL–GPIHBP1 fusion protein is enzymatically stable. The fusion protein was stored at 4 °C for 10 days, and the enzymatic activity was assessed using VLDL as the substrate. The data are plotted relative to activity of fusion protein stored at −80 °C (control). *D*, LPL/GPIHBP1 complex and fusion proteins both have higher specific activity than LPL alone. The ability of the three proteins to hydrolyze TG was assessed using VLDL as the substrate. *mAU*, milli-absorbance units; *RFU*, relative fluorescence units. *Error bars* represent S.D.

GPIHBP1 has been previously reported to stabilize LPL (16, 17). Indeed, we found that addition of the purified soluble fragment of GPIHBPI consisting of residues 21–151 (part of the extracellular domain; Fig. S3*A*) protected LPL against spontaneous inactivation (Fig. S3*B*). We therefore investigated the effect of coexpressing soluble GPIHBP1 (henceforth referred to as GPIHBP1) along with LPL in the presence of LMF1. DNA constructs, encoding N-terminally His_6_-tagged LPL, untagged GPIHBP1, and LMF1 were cotransfected in a ratio of 3:1:1 into HEK293T cells. The expressed protein complex was captured using Ni-affinity chromatography. We found that this triple transfection significantly improved purity and yield of LPL protein (Fig. 1*B*). Importantly, the presence of GPIHBP1 and LMF1 generated an LPL/GPIHBP1 complex that was homogeneous and eluted as an ∼75-kDa complex during size-exclusion chromatography (Fig. 1*D*). This agrees well with the predicted molecular weight of a 1:1 LPL/GPIHBP1 complex. The LPL/GPIHBP1 complex also possessed higher specific activity than LPL alone (Fig. 2*D*) and was resistant to spontaneous inactivation (Fig. 1*E*).

### LPL–GPIHBP1 fusion protein is homogeneous and stable and has high specific activity

The beneficial effect of GPIHBP1 on LPL solubility, activity, and stability prompted us to try to create a nondissociating complex of LPL and GPIHBP1 by making an LPL–GPIHBP1 fusion construct. Mammalian expression vectors were designed that had the LPL and GPIHBP1 open reading frames connected via a 20-amino-acid glycine/serine linker. To aid in purification, we added tags to the N- or C-terminal end of the fusion constructs (Fig. S1). Fig. 2*A* shows the purified LPL-GPIHBP1 fusion with a C-terminal FLAG-His_6_-AviTag (FHA) tag. The fusion protein was obtained with >95% purity after Ni-affinity and size-exclusion chromatography (Fig. 2*A*). Similar to the LPL/GPIHBP1-coexpressed complex, the fusion protein was free of aggregates and resolved as a single homogeneous species with a molecular weight of ∼75 kDa by size-exclusion chromatography (Fig. 2*B*). This indicates that the LPL/GPIHBP1 complex, as well as the fusion protein, consists of one LPL and one GPIHBP1 molecule. These data suggest that LPL exists *in vivo* as a 1:1 LPL/GPIHBP1 complex and not as a head-to-tail dimer as has been suggested previously (18, 19). This 1:1 model also agrees well with the recently solved crystal structure of the coexpressed LPL/GPIHBP1 complex (13). The fusion protein had high enzymatic activity with natural substrates for LPL (VLDL and CM) (Fig. S6, *A* and *B*), was enzymatically stable at 4 °C (Fig. 2*C*), and possessed activity comparable with the copurified LPL/GPIHBP1 complex (Fig. 2*D*), thereby indicating that the fusion does not adversely affect the catalytic activity of LPL.

### ANGPTL4 dissociates LPL/GPIHBP1 complex

It has been reported that GPIHBP1 stabilizes LPL against inactivation by its antagonists, ANGPTL3 and ANGPTL4 (16). ANGPTL4-mediated inactivation of LPL was also reported to dissociate LPL from GPIHBP1 (20). We therefore investigated ANGPTL4/LPL/GPIHBP1 interactions in our assays. We confirmed by several techniques that binding of ANGPTL4 to the LPL/GPIHBP1 complex leads to dissociation of GPIHBP1.

**Figure 3.**
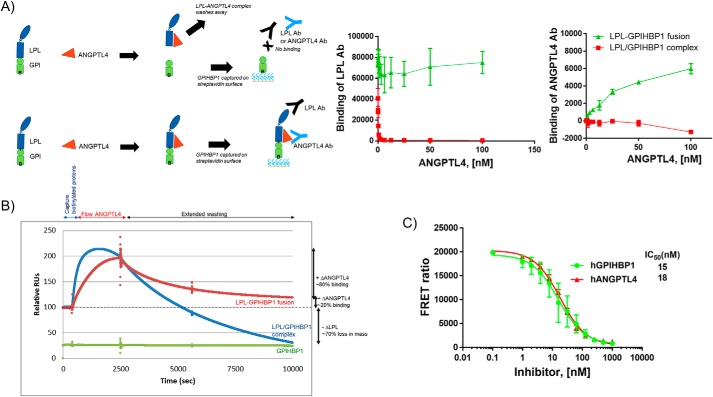
**ANGPTL4 dissociates LPL/GPIHBP1 complex.** *A*, ELISA showing displacement of LPL from the LPL/GPIHBP1 complex by ANGPTL4. A schematic representation of the reaction is drawn on *left* of the graphs. *Left* graph (detection of LPL), ANGPTL4 displaces LPL from LPL/GPIHBP1 complex (*red*) but not the fusion protein (*green*). *Right* graph (detection of ANGPTL4), ANGPTL4 binding to the fusion protein (*green*) but not to the LPL/GPIHBP1 complex (*red*) was observed. *B*, SPR showing displacement of LPL from the LPL/GPIHBP1 complex by ANGPTL4. LPL/GPIHBP1 complex, LPL–GPIHBP1 fusion protein, or GPIHBP1 was immobilized on a streptavidin chip using biotinylated (*B*) AviTag on the C terminus of GPIHBP1. *Red* trace, LPL–GPIHBP1 fusion shows binding of ANGPTL4 to the complex. *Blue* trace, LPL/GPIHBP1 complex is displaced by ANGPTL4. *Green* trace, ANGPTL4 does not bind to GPIHBP1 alone. *C*, FRET assay showing that both ANGPTL4 and GPIHBP1 compete for binding to LPL. LPL(HA)/GPIHBP1(Avi) complex was challenged with increasing concentrations of ANGPTL4 or GPIHBP1 proteins. The FRET signal was generated by using anti-HA-Tb and streptavidin-D2 labels for LPL and GPIHBP1, respectively. The graph shows that ANGPTL4 and free GPIHBP1 dissociated the LPL–GPIHBP1 complex with comparable IC_50_ values. The data were fitted to log(antagonist) *versus* response using a variable-slope (four-parameter) equation using GraphPad Prism. *Ab*, antibody; *RUs*, response units; *h*, human. *Error bars* represent S.D.

First, we showed the dissociation of the complex by ELISA. A schematic representation of the assay is depicted in Fig. 3*A*. Coexpressed LPL/GPIHBP1 complex or the LPL–GPIHBP1 fusion protein were site-specifically biotinylated at an AviTag on the C-terminal end of GPIHBP1 and immobilized on a streptavidin surface. We then incubated the proteins with ANGPTL4 and monitored changes in bound ANGPTL4 and LPL with respective high-affinity antibodies. When the complex was incubated with ANGPTL4, we observed displacement of LPL from the coexpressed LPL/GPIHBP1 complex as a function of ANGPTL4 concentration. In contrast, ANGPTL4 was unable to displace LPL from the covalently linked LPL–GPIHBP1 fusion protein (Fig. 3*A*, *left panel*). Correspondingly, when we probed for bound ANGPTL4, we observed that ANGPTL4 was not captured by the coexpressed LPL/GPIHBP1 complex (ANGPTL4 was displaced along with LPL). In contrast, ANGPTL4 could not displace LPL from the covalent complex and stayed bound to LPL covalently linked to GPIHBP1 (Fig. 3*A*, *right panel*).

We also investigated the interaction of ANGPTL4 with LPL and GPIHBP1 by surface plasmon resonance (SPR) (Fig. 3*B*). We confirmed displacement of LPL by ANGPTL4 from coexpressed LPL/GPIHBP1 complex (*blue* trace) but not from the fusion protein (*red* trace). Finally, in a TR-FRET assay, when we challenged the LPL/GPIHBP1 complex with free GPIHBP1 or ANGPTL4, both proteins were able to dissociate the complex with similar IC_50_ values (Fig. 3*C*), thereby indicating that binding of ANGPTL4 and GPIHBP1 to LPL is mutually exclusive. ANGPTL3 was also able to dissociate the complex, albeit not as efficiently as ANGPTL4 (Fig. S5). Our observations are consistent with functional competition of ANGPTL4 and GPIHBP1 for LPL. Hence, we speculated that the LPL–GPIHBP1 fusion protein may be more resistant to inactivation by ANGPTL4 than either free LPL or the coexpressed LPL/GPIHBP1 complex.

### LPL–GPIHBP1 fusion is resistant to inactivation by ANGPTL4 and ANGPTL3

**Figure 4.**
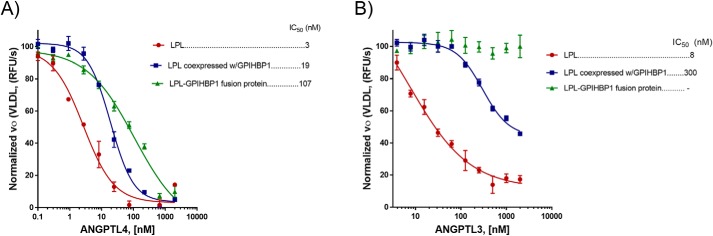
**LPL–GPIHBP1 fusion is resistant to inactivation by ANGPTL4 and ANGPTL3.** *A* and *B*, IC_50_ values of ANGPTL4- and ANGPLT3-mediated inactivation of LPL. The activities of 10 nm LPL (*red*), LPL/GPIHBP1 complex (*blue*), and LPL–GPIHBP1 fusion (*green*) proteins were measured as a function of ANGPTL4 and ANGPTL3 using VLDL as a substrate. The values are plotted related to the activity in the absence of the inhibitors. The data were fit to log(antagonist) *versus* response using a variable-slope (four-parameter) equation using GraphPad Prism. *RFU*, relative fluorescence units. *Error bars* represent S.D.

We investigated the effect of ANGPTL3 and ANGPTL4 on the enzymatic activity of LPL. We observed that the LPL/GPIHBP1-coexpressed complex was significantly more resistant to ANGPTL4 inactivation than LPL alone (IC_50_ values of 19 and 3 nm, respectively) (Fig. 4*A*). Similarly, the LPL/GPIHBP1-coexpressed complex was significantly more resistant to ANGPTL3 inactivation than LPL alone (IC_50_ values of 300 and 8 nm, respectively) (Fig. 4*B*). This indicated that GPIHBP1 not only protects LPL from spontaneous inactivation but also stabilizes it against inactivation by ANGPTLs. This stabilizing effect of GPIHBP1 was even more pronounced when fused to LPL in the LPL–GPIHBP1 fusion protein. The IC_50_ for ANGPTL4-mediated inactivation of the fusion protein was 36-fold higher than that for inactivation of LPL alone and 6-fold higher than for inactivation of the coexpressed LPL/GPIHBP1 complex (Fig. 4*A*). The stabilizing effect of GPIHBP1 against ANGPTL3 inactivation was also more prominent for the fusion protein; we observed no loss of activity by the LPL–GPIHBP1 fusion at ANGPTL3 concentrations up to 2000 nm (Fig. 4*B*).

### LPL site involved in ANGPTL4 binding was successfully mapped by hydrogen–deuterium exchange (HDX) mass spectrometry (MS)

**Figure 5.**
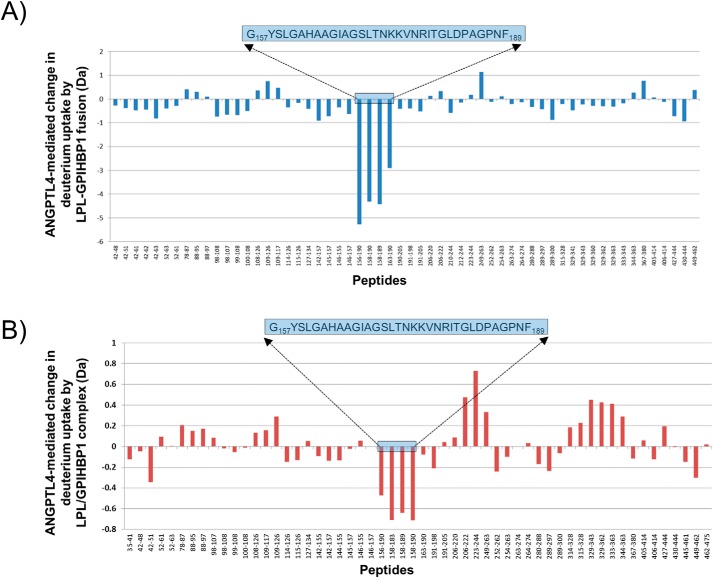
**ANGPTL4-binding site on LPL mapped by HDX-MS.** *A* and *B*, ANGPTL4-binding site map using LPL–GPIHBP1 fusion and LPL/GPIHBP1 complex, respectively. The common region protected by ANGPTL4 in both constructs is marked with the *blue box* (amino acids 157–189).

Our studies thus far demonstrated that the fusion protein is more resistant than the coexpressed LPL/GPIHBP1 complex. Due to the increased stability of the fusion protein, we used this protein complex to map the ANGPTL4-binding site on LPL using HDX in combination with MS. We identified a sequence of 32 LPL amino acid residues (157–189) in the LPL–GPIHBP1 fusion protein that was shielded by the presence of ANGPTL4 (Fig. 5*A*). The same sequence was also protected from the deuterium exchange in the coexpressed LPL/GPIHBP1 complex (Fig. 5*B*). This suggests that the interaction of LPL and ANGPTL4 is not altered by the fusion of LPL to GPIHBP1.

### LPL–GPIHBP1 fusion protein lowers TG in multiple strains of mice

**Figure 6.**
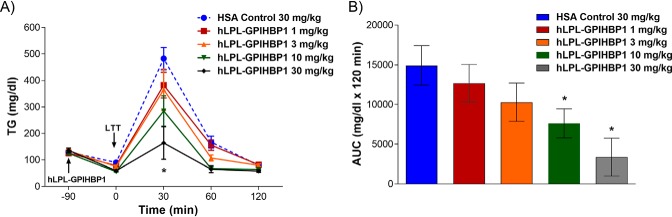
**LPL–GPIHBP1 fusion protein lowers triglycerides in C57BL/6 mice during lipid challenge.** *A*, LPL–GPIHBP1 fusion protein (dosed s.c.; *upward-facing arrow*) mediated lowering of TG after bolus of i.v. Intralipid injection ((lipid tolerance test (*LTT*); *downward-facing arrow*). TG lowering at different concentrations of the fusion protein is plotted as function of time. * indicates *p* < 0.05 for 3, 10 and 30 mg/kg dose versus control (two-way ANOVA). *B*, graph showing 80% AUC reduction at the highest dose of the fusion protein. * indicates *p* < 0.05 versus HSA (one-way ANOVA). *Error bars* represent S.E.

**Figure 7.**
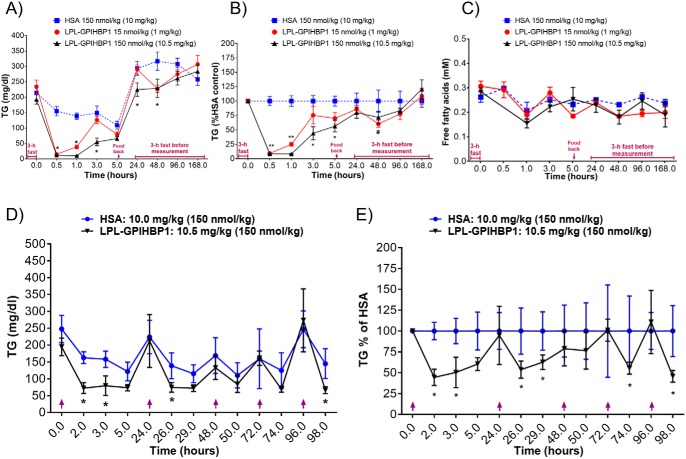
**LPL–GPIHBP1 lowers TG in DBA/2 mice after single as well as repeat dosing.** *A* and *B*, TG lowering in DBA/2 mice after single i.v. dosing of fusion protein. After a 3-h fasting period, mice were dosed i.v. with LPL–GPIHBP1 fusion protein (1 (*red*) and 10.5 mg/kg (*black*)) or HSA (10 mg/kg control, *blue*). Blood was drawn at times indicated on *x axis. A*, absolute TG values. * indicates *p* < 0.05 versus control (two-way ANOVA). *B*, TG values expressed as a percentage relative to HSA control. #, * and ** indicates *p* < 0.05 for the marked dose versus control (two-way ANOVA). *C*, FFA levels monitored after i.v. dosing of fusion protein. *D* and *E*, TG lowering in DBA/2 mice after repeated s.c. dosing of fusion protein. HSA (10 mg/kg; *blue circle*s) and LPL–GPIHBP1 fusion protein (10.5 mg/kg; *black inverted triangles*) were dosed repeatedly at 0, 24, 48, 72, and 96 h (*purple upward-facing arrows*). Blood was drawn at times indicated on *x axis. D*, absolute TG values. *E*, TG values expressed as a percentage relative to HSA control. * indicates *p* < 0.05 versus HSA (two-way ANOVA). *Error bars* represent S.E.

**Figure 8.**
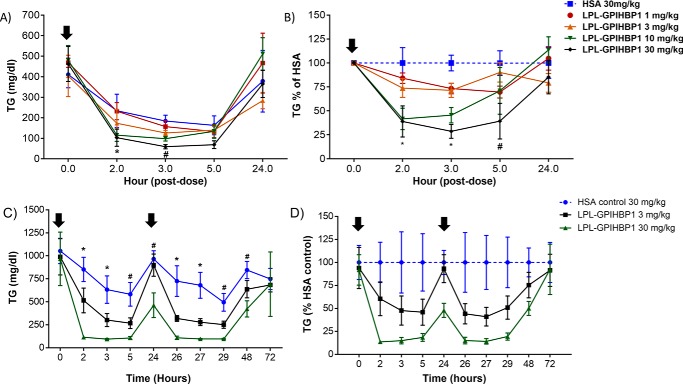
**s.c. administered LPL–GPIHBP1 fusion protein lowers TG in TALLYHO mice on regular as well as high-fat/high-sucrose diet.** *A* and *B*, TG lowering in TALLYHO mice on regular diet after single s.c. dosing of fusion protein. Mice were injected (*black downward-facing arrow*) with the indicated doses of LPL–GPIHBP1 fusion protein or HSA (control; *blue*). Blood was drawn at the times indicated on *x axis. A*, absolute TG values. *B*, TG values expressed as a percentage relative to HSA control. *C* and *D*, TG lowering in TALLYHO mice on high-fat/high-sucrose diet after repeat s.c. dosing of fusion protein. Mice were injected (*black downward-facing arrows*) with the indicated doses of LPL–GPIHBP1 fusion protein or HSA (control; *blue*). Blood was drawn at the times indicated on *x axis. C*, absolute TG values. *D*, TG values expressed as a percentage relative to HSA control. *Error bars* represent S.E.

We expected that the LPL–GPIHBP1 fusion protein would effectively lower plasma TG levels and tested this in a number of mouse strains exhibiting a range of baseline TG levels. We first tested TG lowering in C57BL/6 mice. Because these mice have intrinsically low TG levels (∼100 mg/dl), we transiently increased their plasma TG with a bolus of Intralipid (lipid tolerance test; Fig. 6). As the figure indicates, this induced a ∼5-fold increase in plasma TG levels ∼30 min after injection of the Intralipid bolus (Fig. 6, *blue dashed line*). Subcutaneous administration of LPL–GPIHBP1 dose-dependently blunted the TG increase, with the highest dose lowering the area under the curve (AUC) of the TG excursion by ∼80% (Fig. 6, *A* and *B*). Next, we tested the effect of the fusion protein on TG levels in DBA/2 mice, which have baseline plasma TG concentrations of ∼200 mg/dl. Because TG levels in mouse plasma vary substantially over the course of the day, decreasing by 50–60% during the nonfeeding period, we normalized the fusion protein–mediated reduction in TG levels to the TG levels observed in mice administered an inactive control protein (human serum albumin (HSA)). LPL–GPIHBP1 administration dose-dependently lowered TG in DBA/2 mice after i.v. administration with >90% TG reduction observed with the highest dose (Fig. 7, *A* and *B*). One concern with rapid TG lowering of such magnitude is the possibility of an increase in proinflammatory free fatty acids in plasma. We did not observe any increase in plasma free fatty acids (Fig. 7*C*), suggesting that the free fatty acids were taken up and utilized by surrounding tissues. In a subsequent experiment, we administered LPL–GPIHBP1 to DBA/2 mice daily for 5 days and observed a consistent suppression of plasma TG (Fig. 7, *D* and *E*) without overt TG accumulation in the liver, heart, skeletal muscle, or adipose tissue (Fig. S6). In response to a lipid challenge, LPL–GPIHBP1 also dose-dependently lowered plasma TG in the DBA/2 mice, similar to the response observed in C57BL/6 mice. Subcutaneous administration of LPL–GPIHBP1 dose-dependently blunted the TG increase, with the highest dose lowering the AUC of the TG excursion by ∼90% (Fig. S7, *A* and *B*). Finally, we tested the effect of LPL–GPIHBP1 on TG lowering in hyperlipidemic TALLYHO mice, with baseline TG of ∼400 mg/dl. Subcutaneous administration of LPL–GPIHBP1 dose-dependently lowered TG, with the highest dose lowering TG by ∼70% (Fig. 8, *A* and *B*). In high fat/high sucrose–fed TALLYHO mice with TG baseline ∼1000 mg/dl, repeat s.c. administration of LPL–GPIHBP1 dose-dependently decreased plasma TG, with the highest dose resulting in ∼90% TG lowering (Fig. 8, *C* and *D*). These data convincingly demonstrate that the LPL–GPIHBP1 fusion protein can acutely lower TG *in vivo*.

## Discussion

There are two distinct forms of chylomicronemia. The first form is a rare monogenic disorder known as FCS. It presents in childhood or adolescence and is caused by loss of functional LPL. In ∼80% of the cases, FCS is caused by mutations in LPL itself. Less often, FCS is due to mutations in LPL cofactors and interacting proteins, including GPIHBP1, APOA5, APOC2, or LMF1 (21). Regardless of the underlying mutation, patients with monogenic FCS are phenotypically similar (21). It is estimated that the prevalence of FCS is 1 in 250,000–1,000,000 in the general population (22).

The second form, polygenic late-onset chylomicronemia, is caused by an accumulation of several genetic variants. It can be exacerbated by secondary factors, including poor diet, obesity, alcohol intake, or uncontrolled type 1 or type 2 diabetes mellitus. It is more common than early-onset chylomicronemia. The presence of chylomicrons during fasting is associated with fasting triglyceride levels >10 mmol/liter (885 mg/dl). Using this surrogate triglyceride-cutoff level, primary chylomicronemia has been estimated to occur in ∼1:600 adults in the general population (22).

Taking into account the United States population of 328 million, the prevalence translates into ∼550,000 United States residents having chylomicronemia syndrome, including 300–1,300 patients living with FCS. Both forms of chylomicronemia are associated with increased risk of life-threatening pancreatitis (21–23). In addition to the physical and emotional toll on patients and their families, HTAP is associated with significant healthcare costs. An average episode of HTAP generates medical costs of more than $30,000, which are almost entirely due to inpatient care (9).

Presently, there is no approved pharmacological intervention for the treatment of HTAP. Because LPL deficiency is the underlying cause of FCS, LPL supplementation is expected to alleviate the disease. The LPL gene therapy alipogene tiparvovec was approved in the European Union under the trade name Glybera in 2012. However, it was not successful due to the difficulty of administration, poor efficacy, and high cost. Glybera never attained marketing authorization in the United States and was recently withdrawn from the market in the European Union. LPL supplementation via administration of exogenously expressed LPL has not been pursued in the clinic because LPL is a difficult protein to express and purify, and the purified protein is highly aggregated and enzymatically unstable.

In this study, we report observations that shed light on basic LPL biology as well as remove some of the major roadblocks to the treatment of chylomicronemia with LPL enzyme replacement therapy. It was generally accepted that LPL is active as the head-to-tail dimer and that LPL monomers are catalytically inert. In contradiction to this dogma, we recently showed that LPL monomers form 1:1 enzymatically active complexes with GPIHBP1 and solved the crystal structure of this complex (13). In the present study, we provide further evidence to show that LPL/GPIHBP1 complex is fully active and stable. Even though our system is nonphysiological (we coexpressed LPL and GPIHBP1 in the same cell, whereas *in vivo* the two proteins are expressed by parenchymal and endothelial cells, respectively, and form a complex during transcytosis), our observations are consistent with the recent publication. Our crystal structure of the LPL/GPIHBP1 complex agrees well with the one formed by mixing the two proteins purified separately (19). Also, a paper published while this manuscript was in preparation reported LPL activity as a monomer (24).

By coexpressing LPL and GPIHBP1 in the presence of LMF1, we improved LPL expression and purification yields and generated an LPL/GPIHBP1 complex that was not prone to aggregation (Fig. 1, *B* and *D*). This LPL/GPIHBP1 complex also increased resistance of LPL to spontaneous inactivation as well as inactivation by the LPL antagonists ANGPTL3 and ANGPTL4 (Figs. 1*E* and 4). We showed that GPIHBP1 stabilizes LPL by competing with ANGPTL3 and ANGPTL4 for binding to the enzyme (Figs. 3 and S5). We further mapped LPL amino acids involved in binding to ANGPTL4, the most potent LPL antagonist (Fig. 5). High enzymatic activity of the complex toward LPL natural substrates, high stability, and resistance to inactivation suggest that the monomeric LPL/GPIHBP1 complex may represent a functional enzymatic unit *in vivo*. Taking advantage of observed beneficial effects of GPIHBP1, we “locked” LPL in the protective complex by generating an LPL–GPIHBP1 fusion protein. The fusion protein maintained all the benefits of LPL/GPIHBP1 coexpression: high yields, resistance to aggregation, high enzymatic activity, and resistance to inactivation by ANGPTL3 and ANGPL4.

Given the high enzymatic activity and acceptable pharmacological properties of the LPL–GPIHBP1 fusion protein, we tested its ability to lower TG *in vivo* with the goal of developing it as a drug for the treatment and prevention of HTAP. We tested LPL–GPIHBP1 in C57BL/6, DBA2, and TALLYHO mice that have fasting TG levels of ∼100, ∼200, and ∼400 mg/dl, respectively. In an attempt to mimic severe hypertriglyceridemia, we maintained TALLYHO mice on a high-fat/high-sucrose diet, which increased their TG to ∼1000 mg/dl. We also subjected mice to an oral lipid challenge, which transiently increased their TG severalfold. We saw significant dose-dependent TG lowering in all strains after single or repeat i.v. and s.c. dosing (Figs. 6–8). At high doses, LPL–GPIHBP1 lowered TG by >95%. A potential concern is that massive TG hydrolysis may lead to a significant increase in proinflammatory free fatty acids (FFA) at the site of injection and/or in plasma. That does not appear to be the case. More than 90% lipid lowering by LPL–GPIHBP1 during oral lipid challenge did not lead to an increase in plasma FFA (Fig. 7*C*). We also saw no injection site reactions in any of the *in vivo* studies. This suggests that liberated FFA are efficiently taken up by surrounding tissues. We also did not observe an increase in hepatic, intramuscular, or cardiac fat or significant adipose tissue expansion (Fig. S6). This suggests that FFA were most likely utilized as an energy source. Further studies will be needed to confirm these results.

LPL–GPIHBP1 has the potential to be effective in treating and preventing abdominal pain and hyperlipidemic pancreatitis by acutely lowering plasma TG. Administration of LPL–GPIHBP1 at the first signs of impending HTAP might relieve abdominal pain and stave off a full-blown HTAP attack. LPL–GPIHBP1 treatment may also maintain TG in the safe range long-term. The extremely high TG in FCS are of alimentary origin. Clinical experience has demonstrated that rapid TG lowering by plasmapheresis permits maintenance of TG in a safe range for several days when TG influx is concomitantly limited by a low-fat diet. Based on these results, we expect that TG lowering by LPL–GPIHBP1 will maintain TG in the safe range for patients with extremely high TG.

## Experimental procedures

### Enzymes and reagents

Amplex Red and resorufin butyrate were purchased from Life Technologies. Human VLDL and CM were from EMD Millipore and Athens Research and Technology, respectively. BSA was obtained from Sigma. The HR Series NEFA-HR(2) Color Reagent A and HR Series NEFA-HR(2) Color Reagent B were purchased from Wako Diagnostics. Detergents were obtained from Sigma.

### Expression plasmids

Mammalian expression vectors for LPL, GPIHBP1, LPL–GPIHBP1 fusion, ANGPTL3, and ANGPTL4 were synthesized by Life Technologies. The sequences of the open reading frames are listed in Table 1.

**Table 1:** **Amino acid sequences of recombinant human LPL, soluble human (h) GPIHBP1, human ANGPTL3, human ANGPTL4, human ANGPTL3, and human LPL–GPIHBP1 fusion proteins** Purification tags are highlighted with italics. TEV, tobacco etch virus; (G4S)4, (GGGGS)_4_. TEV cleavage sequence are indicated by bold and His_6_ are indicated by underline.

Recombinant protein	Amino acid sequence
LPL	ADQRRDFIDIESKFALRTPEDTAEDTCHLIPGVAESVATCHFNHSSKTFMVIHGWTVTGMYESWVPKLVAALYKREPDSNVIVVDWLSRAQEHYPVSAGYTKLVGQDVARFINWMEEEFNYPLDNVHLLGYSLGAHAAGIAGSLTNKKVNRITGLDPAGPNFEYAEAPSRLSPDDADFVDVLHTFTRGSPGRSIGIQKPVGHVDIYPNGGTFQPGCNIGEAIRVIAERGLGDVDQLVKCSHERSIHLFIDSLLNEENPSKAYRCSSKEAFEKGLCLSCRKNRCNNLGYEINKVRAKRSSKMYLKTRSQMPYKVFHYQVKIHFSGTESETHTNQAFEISLYGTVAESENIPFTLPEVSTNKTYSFLIYTEVDIGELLMLKLKWKSDSYFSWSDWWSSPGFAIQKIRVKAGETQKKVIFCSREKVSHLQKGKAPAVFVKCHDKSLNKKSG
His_6_-**TEV**-LPL	*HHHHHH***ENLYFQGA**DQRRDFIDIESKFALRTPEDTAEDTCHLIPGVAESVATCHFNHSSKTFMVIHGWTVTGMYESWVPKLVAALYKREPDSNVIVVDWLSRAQEHYPVSAGYTKLVGQDVARFINWMEEEFNYPLDNVHLLGYSLGAHAAGIAGSLTNKKVNRITGLDPAGPNFEYAEAPSRLSPDDADFVDVLHTFTRGSPGRSIGIQKPVGHVDIYPNGGTFQPGCNIGEAIRVIAERGLGDVDQLVKCSHERSIHLFIDSLLNEENPSKAYRCSSKEAFEKGLCLSCRKNRCNNLGYEINKVRAKRSSKMYLKTRSQMPYKVFHYQVKIHFSGTESETHTNQAFEISLYGTVAESENIPFTLPEVSTNKTYSFLIYTEVDIGELLMLKLKWKSDSYFSWSDWWSSPGFAIQKIRVKAGETQKKVIFCSREKVSHLQKGKAPAVFVKCHDKSLNKKSG
LPL-FHA	ADQRRDFIDIESKFALRTPEDTAEDTCHLIPGVAESVATCHFNHSSKTFMVIHGWTVTGMYESWVPKLVAALYKREPDSNVIVVDWLSRAQEHYPVSAGYTKLVGQDVARFINWMEEEFNYPLDNVHLLGYSLGAHAAGIAGSLTNKKVNRITGLDPAGPNFEYAEAPSRLSPDDADFVDVLHTFTRGSPGRSIGIQKPVGHVDIYPNGGTFQPGCNIGEAIRVIAERGLGDVDQLVKCSHERSIHLFIDSLLNEENPSKAYRCSSKEAFEKGLCLSCRKNRCNNLGYEINKVRAKRSSKMYLKTRSQMPYKVFHYQVKIHFSGTESETHTNQAFEISLYGTVAESENIPFTLPEVSTNKTYSFLIYTEVDIGELLMLKLKWKSDSYFSWSDWWSSPGFAIQKIRVKAGETQKKVIFCSREKVSHLQKGKAPAVFVKCHDKSLNKKSG*DYKDDDDKHHHHHHGGGLNDIFEAQKIEWHE*
His_6_-LPL-**HA**-FLAG	*HHHHHH*ADQRRDFIDIESKFALRTPEDTAEDTCHLIPGVAESVATCHFNHSSKTFMVIHGWTVTGMYESWVPKLVAALYKREPDSNVIVVDWLSRAQEHYPVSAGYTKLVGQDVARFINWMEEEFNYPLDNVHLLGYSLGAHAAGIAGSLTNKKVNRITGLDPAGPNFEYAEAPSRLSPDDADFVDVLHTFTRGSPGRSIGIQKPVGHVDIYPNGGTFQPGCNIGEAIRVIAERGLGDVDQLVKCSHERSIHLFIDSLLNEENPSKAYRCSSKEAFEKGLCLSCRKNRCNNLGYEINKVRAKRSSKMYLKTRSQMPYKVFHYQVKIHFSGTESETHTNQAFEISLYGTVAESENIPFTLPEVSTNKTYSFLIYTEVDIGELLMLKLKWKSDSYFSWSDWWSSPGFAIQKIRVKAGETQKKVIFCSREKVSHLQKGKAPAVFVKCHDKSLNKKSG**YPYDVPDYA***DYKDDDDK*
Soluble GPIHBP1(21–151)	QTQQEEEEEDEDHGPDDYDEEDEDEVEEEETNRLPGGRSRVLLRCYTCKSLPRDERCNLTQNCSHGQTCTTLIAHGNTESGLLTTHSTWCTDSCQPITKTVEGTQVTMTCCQSSLCNVPPWQSSRVQDPTG
Soluble GPIHBP1(21–151)- FLAG-HIS_6_-Avi	QTQQEEEEEDEDHGPDDYDEEDEDEVEEEETNRLPGGRSRVLLRCYTCKSLPRDERCNLTQNCSHGQTCTTLIAHGNTESGLLTTHSTWCTDSCQPITKTVEGTQVTMTCCQSSLCNVPPWQSSRVQDPTG*DYKDDDDKHHHHHHGGGLNDIFEAQKIEWHE*
ANGPTL4(26–406)-FLAG-His_6_-Avi	QPGPVQSKSPRFASWDEMNVLAHGLLQLGQGLREHAERTRSQLSALERRLSACGSACQGTEGSTDLPLAPESRVDPEVLHSLQTQLKAQNSRIQQLFHKVAQQQRHLEKQHLRIQHLQSQFGLLDHKHLDHEVAKPARRKRLPEMAQPVDPAHNVSRLHRLPRDCQELFQVGERQSGLFEIQPQGSPPFLVNCKMTSDGGWTVIQRRHDGSVDFNRPWEAYKAGFGDPHGEFWLGLEKVHSITGDRNSRLAVQLRDWDGNAELLQFSVHLGGEDTAYSLQLTAPVAGQLGATTVPPSGLSVPFSTWDQDHDLRRDKNCAKSLSGGWWFGTCSHSNLNGQYFRSIPQQRQKLKKGIFWKTWRGRYYPLQATTMLIQPMAAEAAS*DYKDDDDKHHHHHHGGGLNDIFEAQKIEWHE*
ANGPTL3(17–460)-FLAG-His_6_-Avi	WVIFLLPGATAQPSRIDQDNSSFDSLSPEPKSRFAMLDDVKILANGLLQLGHGLKDFVHKTKGQINDIFQKLNIFDQSFYDLSLQTSEIKEEEKELRRTTYKLQVKNEEVKNMSLELNSKLESLLEEKILLQQKVKYLEEQLTNLIQNQPETPEHPEVTSLKTFVEKQDNSIKDLLQTVEDQYKQLNQQHSQIKEIENQLRRTSIQEPTEISLSSKPRAPRTTPFLQLNEIRNVKHDGIPAECTTIYNRGEHTSGMYAIRPSNSQVFHVYCDVISGSPWTLIQHRIDGSQNFNETWENYKYGFGRLDGEFWLGLEKIYSIVKQSNYVLRIELEDWKDNKHYIEYSFYLGNHETNYTLHLVAITGNVPNAIPENKDLVFSTWDHKAKGHFNCPEGYSGGWWWHDECGENNLNGKYNKPRAKSKPERRRGLSWKSQNGRLYSIKSTKMLIHPTDSESFEDYKDDDDKHHHHHHGGGLNDIFEAQKIEWHE
Avi-His_6_-FLAG-LPL(28–475)-(G4S)4-hGPIHBP1(21–151)	GGGLNDIFEAQKIEWHEHHHHHHDYKDDDDKADQRRDFIDIESKFALRTPEDTAEDTCHLIPGVAESVATCHFNHSSKTFMVIHGWTVTGMYESWVPKLVAALYKREPDSNVIVVDWLSRAQEHYPVSAGYTKLVGQDVARFINWMEEEFNYPLDNVHLLGYSLGAHAAGIAGSLTNKKVNRITGLDPAGPNFEYAEAPSRLSPDDADFVDVLHTFTRGSPGRSIGIQKPVGHVDIYPNGGTFQPGCNIGEAIRVIAERGLGDVDQLVKCSHERSIHLFIDSLLNEENPSKAYRCSSKEAFEKGLCLSCRKNRCNNLGYEINKVRAKRSSKMYLKTRSQMPYKVFHYQVKIHFSGTESETHTNQAFEISLYGTVAESENIPFTLPEVSTNKTYSFLIYTEVDIGELLMLKLKWKSDSYFSWSDWWSSPGFAIQKIRVKAGETQKKVIFCSREKVSHLQKGKAPAVFVKCHDKSLNKKSGGGGGSGGGGSGGGGSGGGGSQTQQEEEEEDEDHGPDDYDEEDEDEVEEEETNRLPGGRSRVLLRCYTCKSLPRDERCNLTQNCSHGQTCTTLIAHGNTESGLLTTHSTWCTDSCQPITKTVEGTQVTMTCCQSSLCNVPPWQSSRVQDPTG
LPL(28–475)-(G4S)4-hGPIHBP1(21–151)-FLAG-His_6_-Avi	ADQRRDFIDIESKFALRTPEDTAEDTCHLIPGVAESVATCHFNHSSKTFMVIHGWTVTGMYESWVPKLVAALYKREPDSNVIVVDWLSRAQEHYPVSAGYTKLVGQDVARFINWMEEEFNYPLDNVHLLGYSLGAHAAGIAGSLTNKKVNRITGLDPAGPNFEYAEAPSRLSPDDADFVDVLHTFTRGSPGRSIGIQKPVGHVDIYPNGGTFQPGCNIGEAIRVIAERGLGDVDQLVKCSHERSIHLFIDSLLNEENPSKAYRCSSKEAFEKGLCLSCRKNRCNNLGYEINKVRAKRSSKMYLKTRSQMPYKVFHYQVKIHFSGTESETHTNQAFEISLYGTVAESENIPFTLPEVSTNKTYSFLIYTEVDIGELLMLKLKWKSDSYFSWSDWWSSPGFAIQKIRVKAGETQKKVIFCSREKVSHLQKGKAPAVFVKCHDKSLNKKSGGGGGSGGGGSGGGGSGGGGSQTQQEEEEEDEDHGPDDYDEEDEDEVEEEETNRLPGGRSRVLLRCYTCKSLPRDERCNLTQNCSHGQTCTTLIAHGNTESGLLTTHSTWCTDSCQPITKTVEGTQVTMTCCQSSLCNVPPWQSSRVQDPTGDYKDDDDKHHHHHHGGGLNDIFEAQKIEWHE
His_6_-LPL(28–475)-**HA**-(G4S)4-hGPIHBP1(21–151)-Avi	HHHHHHADQRRDFIDIESKFALRTPEDTAEDTCHLIPGVAESVATCHFNHSSKTFMVIHGWTVTGMYESWVPKLVAALYKREPDSNVIVVDWLSRAQEHYPVSAGYTKLVGQDVARFINWMEEEFNYPLDNVHLLGYSLGAHAAGIAGSLTNKKVNRITGLDPAGPNFEYAEAPSRLSPDDADFVDVLHTFTRGSPGRSIGIQKPVGHVDIYPNGGTFQPGCNIGEAIRVIAERGLGDVDQLVKCSHERSIHLFIDSLLNEENPSKAYRCSSKEAFEKGLCLSCRKNRCNNLGYEINKVRAKRSSKMYLKTRSQMPYKVFHYQVKIHFSGTESETHTNQAFEISLYGTVAESENIPFTLPEVSTNKTYSFLIYTEVDIGELLMLKLKWKSDSYFSWSDWWSSPGFAIQKIRVKAGETQKKVIFCSREKVSHLQKGKAPAVFVKCHDKSLNKKSGYPYDVPDYAGGGGSGGGGSGGGGSGGGGSQTQQEEEEEDEDHGPDDYDEEDEDEVEEEETNRLPGGRSRVLLRCYTCKSLPRDERCNLTQNCSHGQTCTTLIAHGNTESGLLTTHSTWCTDSCQPITKTVEGTQVTMTCCQSSLCNVPPWQSSRVQDPTGGGGLNDIFEAQKIEWHE

### Expression and purification of recombinant proteins

#### 

##### Human LPL

Plasmid encoding full-length human LPL polypeptide (matching NCBI sequence NM_000237.2) was transiently transfected into HEK293T cells using standard polyethylenimine (PEI) transfection methods. Cells were propagated in suspension culture in Freestyle 293 expression medium, and transfection was carried out at 1 × 10^6^ cells/ml final cell concentration. At 24 h after transfection, heparin was added to the culture medium to a final concentration of 3 units/ml to enhance release of secreted LPL from the cell surface. At 60 h post-transfection, the culture medium was collected and filtered using a 0.2-μm filter, and glycerol was added to a final concentration of 10% (v/v). The resulting solution was loaded onto a 5 ml Heparin-Sepharose HiTrap column (GE Healthcare) that had been pre-equilibrated with buffer composed of 50 mm Tris-HCl (pH 7.2), 200 mm NaCl, and 10% (v/v) glycerol. The column was washed with the same buffer until baseline absorbance at 280 nm was reached. LPL was then eluted with step gradients of 500 mm NaCl, 1 m NaCl, and 2 m NaCl. LPL enzymatic activity was measured in the elution fractions, and protein purity was assessed by SDS-PAGE. The most catalytically active and highest-purity LPL eluted with 1 m NaCl. Aliquots of purified human LPL were flash frozen and stored at −80 °C until use.

##### Soluble human GPIHBP1

Plasmid encoding the soluble domain of GPIHBP1 with C-terminal FHA tag was transiently transfected into HEK293T cells using standard PEI transfection methods. Cells were propagated in suspension culture in Freestyle 293 expression medium, and transfection was carried out at 1 × 10^6^ cells/ml final cell concentration. At 60 h post-transfection, cells were harvested by centrifugation followed by filtration with a 0.22-μm filter. The clarified supernatant was concentrated and exchanged into buffer containing 50 mm Tris-HCl (pH 8.0), 150 mm NaCl, 10% (v/v) glycerol, and 20 mm imidazole using tangential-flow filtration (TFF). The concentrated sample was passed over a 5-ml Ni-NTA affinity column (GE Healthcare) equilibrated with buffer containing 50 mm Tris-HCl (pH 8.0), 150 mm NaCl, 10% (v/v) glycerol, and 20 mm imidazole. After loading the sample, the column was washed with the same buffer until baseline absorbance at 280 nm was reached. The bound GPIHBP1 protein was then eluted by running a gradient of imidazole (20–500 mm). Relevant fractions were pooled, concentrated using an Amicon concentrator (molecular-weight cutoff, 10,000 Da), buffer-exchanged using PD-10 columns into storage buffer (PBS), aliquoted, and flash frozen in liquid nitrogen before storage at −80 °C.

##### Human LPL/GPIHBP1 complex

Plasmids encoding human LPL (untagged or with either His or FHA purification tags at the N- or C-terminal end) (hLPL), soluble human GPIHBP1 (untagged or with FHA purification tags at the C-terminal end), and human LMF1 were transiently transfected into suspension-adapted HEK293T cells using a standard PEI transfection method in a molar ratio of 3:1:1. The cells were propagated in suspension culture in Freestyle 293 expression medium, transfected at 1 × 10^6^ cells/ml final cell concentration, and maintained at 37 °C and 5% CO_2_ in a shaking incubator for 72 h. The cells were then harvested by centrifugation, and the supernatant was filtered through a 0.22-μm sterile filter. The clarified supernatant was concentrated and buffer-exchanged into 20 mm Tris-HCl (pH 7.5) containing 500 mm NaCl, 10% (v/v) glycerol, and 20 mm imidazole using TFF. The concentrated sample was then applied to a Ni-NTA affinity column equilibrated with 20 mm Tris-HCl (pH 7.5) containing 500 mm NaCl, 10% (v/v) glycerol, and 20 mm imidazole, and the column was washed with the same buffer until baseline absorbance at 280 nm was reached. The bound hLPL/GPIHBP1 complex was then eluted by running a gradient of imidazole (20–500 mm imidazole in 20 mm Tris-HCl (pH 7.5) containing 500 mm NaCl and 10% (v/v) glycerol), and hLPL/GPIHBP1 complex–containing fractions (identified by SDS-PAGE) were pooled, concentrated (Amicon concentrator; molecular-weight cutoff, 30 kDa), and loaded onto a Superdex 200 16/60 sizing column equilibrated with running buffer (10 mm Tris (pH 7.5) containing 300 mm NaCl). Peak fractions were analyzed by SDS-PAGE, and fractions containing LPL/soluble GPIHBP1 complex were pooled, concentrated, aliquoted, flash frozen in liquid nitrogen, and stored at −80 °C.

##### Human LPL–GPIHBP1 fusion proteins

Plasmids encoding human LPL–(GGGGS)_4_ linker–human GPIHBP1 (with purification tags at the N- or C-terminal end) and human LMF1 were transiently transfected into suspension-adapted HEK293T cells using a standard PEI transfection method in molar ratio of 3:1. The cells were propagated in suspension culture in Freestyle 293 expression medium, transfected at 1 × 10^6^ cells/ml final cell concentration, and maintained at 37 °C and 5% CO_2_ in a shaking incubator for 72 h. The cells were then harvested by centrifugation, and the supernatant was filtered through a 0.22-μm sterile filter. The clarified supernatant was concentrated and buffer-exchanged into 50 mm HEPES (pH 7.3) containing 300 mm NaCl, 10% (v/v) glycerol and 30 mm imidazole using TFF. The concentrated sample was then applied to a HiTrap Ni-affinity column (GE Healthcare) equilibrated with the same buffer, and the column was washed until baseline absorbance at 280 nm was reached. The bound fusion protein was then eluted by running a gradient of imidazole (30–300 mm imidazole in 50 mm HEPES (pH 7.3) containing 300 mm NaCl, 10% (v/v) glycerol), and fractions containing fusion protein (identified by SDS-PAGE) were pooled, concentrated (Amicon concentrator; molecular-weight cutoff, 30 kDa), and loaded onto a Superdex 200 16/60 sizing column (GE Healthcare) equilibrated with 50 mm HEPES (pH 7.3) containing 300 mm NaCl and 10% glycerol. Peak fractions were analyzed by SDS-PAGE, and fractions containing LPL–GPIHBP1 fusion protein were pooled, concentrated, aliquoted, flash frozen in liquid nitrogen, and stored at −80 °C.

### Site-specific biotinylation of proteins

Proteins with AviTag were biotinylated as follows. Purified protein in 50 mm Bicine buffer (pH 8.3) at a final concentration of ∼1 mg/ml was incubated in the presence of 10 mm ATP, 10 mm magnesium acetate, 0.1 mm biotin, and BirA biotin ligase (Avidity) at 30 °C for 1 h and then placed at 4 °C overnight. The protein was then concentrated using an Amicon concentrator (molecular-weight cutoff, 10,000 Da), buffer-exchanged using PD-10 columns into storage buffer (50 mm Tris-HCl (pH 7.4), 150 mm NaCl, and 15% (v/v) glycerol), aliquoted, and flash frozen in liquid nitrogen.

### Biochemical Assays

#### 

##### LPL enzymatic activity with VLDL and CM substrates

The following protocol was used to assess activity. Purified LPL protein (either LPL alone, LPL copurified with GPIHBP1, or LPL–GPIHBP1 fusion) (20 μl/well, diluted in PBS) was mixed with equal volumes of human VLDL or CM (20 μl/well, diluted in PBS) in a 384-well Costar black plate. To this mixture, Amplex Red mixture containing a coupled enzyme system (HR series NEFA-HR(2), Wako Diagnostics) (20 μl/well, diluted in PBS) was added, and the fluorescence of resorufin was monitored continuously for 30 min using an Envision multiwell plate reader (PerkinElmer Life Sciences) using excitation and emission wavelengths of 531 and 590 nm, respectively. When stated, a fixed concentration of LPL was preincubated with ANGPTL3 or ANGPTL4 (serially diluted 2-fold using assay buffer) in a volume of 20 μl for 10 min before addition of VLDL. Final assay concentrations were as follows: varying LPL, varying ANGPTL3 or ANGPTL4, 6.25 μg/ml human VLDL or 10 μg/ml human CM, 0.75 mm ATP, 90 μm CoA, 0.5 unit/ml acetyl-CoA oxidase, 1.25 units/ml acyl-CoA synthetase, 1.2 units/ml horseradish peroxidase, and 10 μm Amplex Red. Data analysis was performed using Microsoft Excel and GraphPad Prism software.

##### LPL enzymatic activity with resorufin butyrate substrate

The assay was performed in a 384-well Costar black plate. LPL (10 nm) was preincubated in the absence or presence of GPIHBP1 (12 nm) in PBS for varying amount of times. The ability of the preincubation mixture to hydrolyze resorufin butyrate (9 μm resorufin) was measured using assay buffer consisting of PBS, 1.5% (w/v) BSA, and 0.025% (v/v) Zwittergent. Activity was monitored using an Envision multiwell plate reader using excitation and emission wavelengths of 500 and 593 nm, respectively. The rate of hydrolysis was calculated over the initial linear phase of the reaction. Data analysis was performed using Microsoft Excel and GraphPad Prism software.

##### ELISA for detection of LPL/GPIHBP1 complex disruption by ANGPTL4

LPL/GPIHBP1 complex or LPL–GPIHBP1 fusion (10 nm; site-specifically biotinylated at the C-terminal end of GPIHBP1) was incubated with increasing concentrations of ANGPTL4 (a 12-point 2-fold serial dilution with highest concentration of 100 nm). The reaction mixture was then applied onto a streptavidin-coated Meso Scale Discovery (MSD) plate. The presence of LPL and ANGPTL4 was detected using LPL- or ANGPTL4-specific antibodies, which were subsequently quantified using SULFO-TAG–tagged secondary antibody. To generate a signal, 1× MSD read buffer T was added, and the plate was developed using a Sector Imager 6000 (Meso Scale Discovery).

##### SPR assay for detection of LPL/GPIHBP1 complex disruption by ANGPTL4

LPL/GPIHBP1 complex, LPL–GPIHBP1 fusion protein, or GPIHBP1 (site-specifically biotinylated at the C-terminal end of GPIHBP1) was immobilized on a streptavidin-coated surface at a concentration of 1 nm. ANGPTL4 at a concentration of 10 nm was flowed over the immobilized complex, and the mass of surface-associated proteins was monitored as a function of time using a Biacore T100 instrument.

##### TR-FRET–based assay for detection of LPL/GPIHBP1 complex disruption

The assay for TR-FRET–based detection of LPL/GPIHBP1 complex disruption was carried out in 384-well plates (ProxiPlate 384-well white, PerkinElmer Life Sciences) in a final volume of 20 μl. The composition of the assay buffer was 20 mm HEPES (pH 7.4), 100 mm NaCl, 10% (v/v) heat-inactivated fetal bovine serum, and 5 mm CaCl_2_. First, 4 μl/well 5× His_6_-hLPL-HA-FLAG/biotinylated hGPIHBP1-Avi complex (50 nm stock in assay buffer; 10 nm final concentration) was added to 8 μl/well buffer. Then 4 μl/well 5× nonbiotinylated ANGPTL4, ANGPTL3, or GPIHBP1 (final concentrations of 1–1000 nm) was added, and the mixture was incubated at room temperature for 1 h. The TR-FRET signal was generated by using anti-HA-Tb labels and streptavidin-D2 for LPL and GPIHBP1, respectively. The signal was detected using an Envision plate reader with an excitation wavelength of 320 nm and emission wavelengths of 665 and 615 nm.

##### Epitope mapping by hydrogen–deuterium exchange/MS

HDX-MS (25) was used to map the ANGPTL4-binding epitope on LPL. Automated HDX-MS experiments were performed using methods similar to those described in the literature (26). The experiments were performed on a Waters HDX-MS platform, which includes a LEAP autosampler, nanoACQUITY UPLC system, and Synapt G2 mass spectrometer. The LPL/GPIHBP1 complex or LPL–GPIHBP1 fusion protein (15.8 μm) in the absence or presence of ANGPTL4 (79.2 μm) was labeled in a deuterium Tris-HCl buffer at pH 7.0 for 15 min at 4 °C. The labeling reaction was then quenched with chilled quench buffer on ice for 3 min. Next, the quenched protein solution was injected onto the LC-MS system for automated pepsin digestion and peptide analysis. The ANGPTL4-binding epitope on the complex and fusion was mapped by comparing the LC-MS data in the absence and presence of ANGPTL4. All measurements were carried out using a minimum of three analytical triplicates.

### Animal studies

Male, 12-week-old C57BL/6 (Taconic, Rensselaer, NY), 12–15-week-old DBA/2J, or 15–24-week-old TALLYHO/JngJ mice (The Jackson Laboratory, Bar Harbor, ME) were used for the studies. Animals were housed in normal light cycle (6:00 a.m.–6:00 p.m.), fed on a normal chow or a high-fat and high-sucrose diet (Research Diets catalog number D12331i), and had access to water *ad libitum* during the studies. All procedures were in compliance with the Animal Welfare Act Regulations Title 9, Code of Federal Regulations Parts 1, 2, and 3 and other guidelines. The studies were performed under an animal protocol approved by the Institutional Animal Care and Use Committee of Novartis Institutes for BioMedical Research. Blood samples were taken by tail vein, collected in Microvette tubes (Sarstedt AG & Co., Numbrecht, Germany), and kept on ice before centrifugation. Animals were randomly assigned into either vehicle or treatment groups (*n* = 6–8/group) with serum TG and FFA levels measured using a Wako Diagnostics kit (Mountain View, CA) and matched between groups. On the day of the study, animals were dosed i.v. or s.c. with HSA (as negative control) or LPL–GPIHBP1 in PBS at doses from 0.3 to 30 mg/kg. Tail blood samples were taken before dosing and at multiple time points after dosing. Serum TG levels were determined as described above. Lipid tolerance tests were performed in C57BL/6 and DBA/2J mice by i.v. injection of Intralipid (a phospholipid-stabilized soybean oil as 20% fat emulsion; Sigma-Aldrich). Serum samples were obtained from the tail vein before and at 0.5, 1, and 2 h after Intralipid injection for TG determination.

### Statistical analyses

Statistical analyses were performed using a two-way analysis of variance (ANOVA) followed by a post hoc test using Bonferroni's method for each time point in GraphPad Prism. Data are presented as mean ± S.E. Statistical significance was accepted at the level of *p* < 0.05.

## Author contributions

A. V. N., S. W., J. W. T., J. G., and A. V. conceptualization; A. V. N., S. W., and A. V. data curation; A. V. N., S. W., K. G., D. P., S. H., R. C., F. X., J. G., and A. V. investigation; A. V. N., S. W., K. G., D. P., S. H., R. C., F. X., J. G., and A. V. methodology; A. V. N. and A. V. writing-original draft; A. V. N. and A. V. writing-review and editing; A. V. formal analysis; A. V. supervision; A. V. project administration.

## Supplementary Material

Supporting Information

## Footnotes

2 The abbreviations used are:

LPL: lipoprotein lipase
GPIHBP1: glycosylphosphatidylinositol-anchored high-density lipoprotein–binding protein 1
FCS: familial chylomicronemia syndrome
ANGPTL: angiopoietin-like protein
LMF1: lipase maturation factor 1
TG: triglycerides
VLDL: very-low-density lipoproteins
CM: chylomicrons
HTAP: hypertriglyceridemic pancreatitis
i.v.: intravenous
s.c.: or subcutaneous
FHA: FLAG-His_6_-AviTag
SPR: surface plasmon resonance
TR-FRET: time-resolved FRET
HDX: hydrogen–deuterium exchange
AUC: area under the curve
HSA: human serum albumin
FFA: free fatty acids
PEI: polyethylenimine
TFF: tangential-flow filtration
Ni-NTA: nickel-nitrilotriacetic acid
hLPL: human LPL
Bicine: *N*,*N*-bis(2-hydroxyethyl)glycine
HA: hemagglutinin.
