# Supplementary material for: A lipoprotein lipase–GPI-anchored high-density lipoprotein–binding protein 1 fusion lowers triglycerides in mice: Implications for managing familial chylomicronemia syndrome
Source: J Biol Chem. 2019 Oct 23;295(10):2900–12. doi: 10.1074/jbc.RA119.011079 (PMC7062184; doi:10.1074/jbc.RA119.011079)
Supplement: Supporting Information [file supp_295_10_2900__index.html]

A lipoprotein lipase --GPI-anchored high density lipoprotein binding protein 1 fusion lowers triglycerides in mice: implications for managing familial chylomicronemia syndrome — LPL protein therapy for chylomicronemia — A lipoprotein lipase–GPI-anchored high-density lipoprotein–binding protein 1 fusion lowers triglycerides in mice: Implications for managing familial chylomicronemia syndrome — EDITORS' PICK: LPL protein therapy for chylomicronemia — Supporting Information 

# A lipoprotein lipase–GPI-anchored high-density lipoprotein–binding protein 1 fusion lowers triglycerides in mice: Implications for managing familial chylomicronemia syndrome

## Supporting Information

- Supporting Information (to be published online) - Supplementary data
