# Supplementary material for: A lipoprotein lipase–GPI-anchored high-density lipoprotein–binding protein 1 fusion lowers triglycerides in mice: Implications for managing familial chylomicronemia syndrome
Source: J Biol Chem. 2019 Oct 23;295(10):2900–12. doi: 10.1074/jbc.RA119.011079 (PMC7062184; doi:10.1074/jbc.RA119.011079)
Supplement: Supporting Information [file supp_RA119.011079_155850_1_supp_415387_pzqm4t.pdf]

Supporting Information

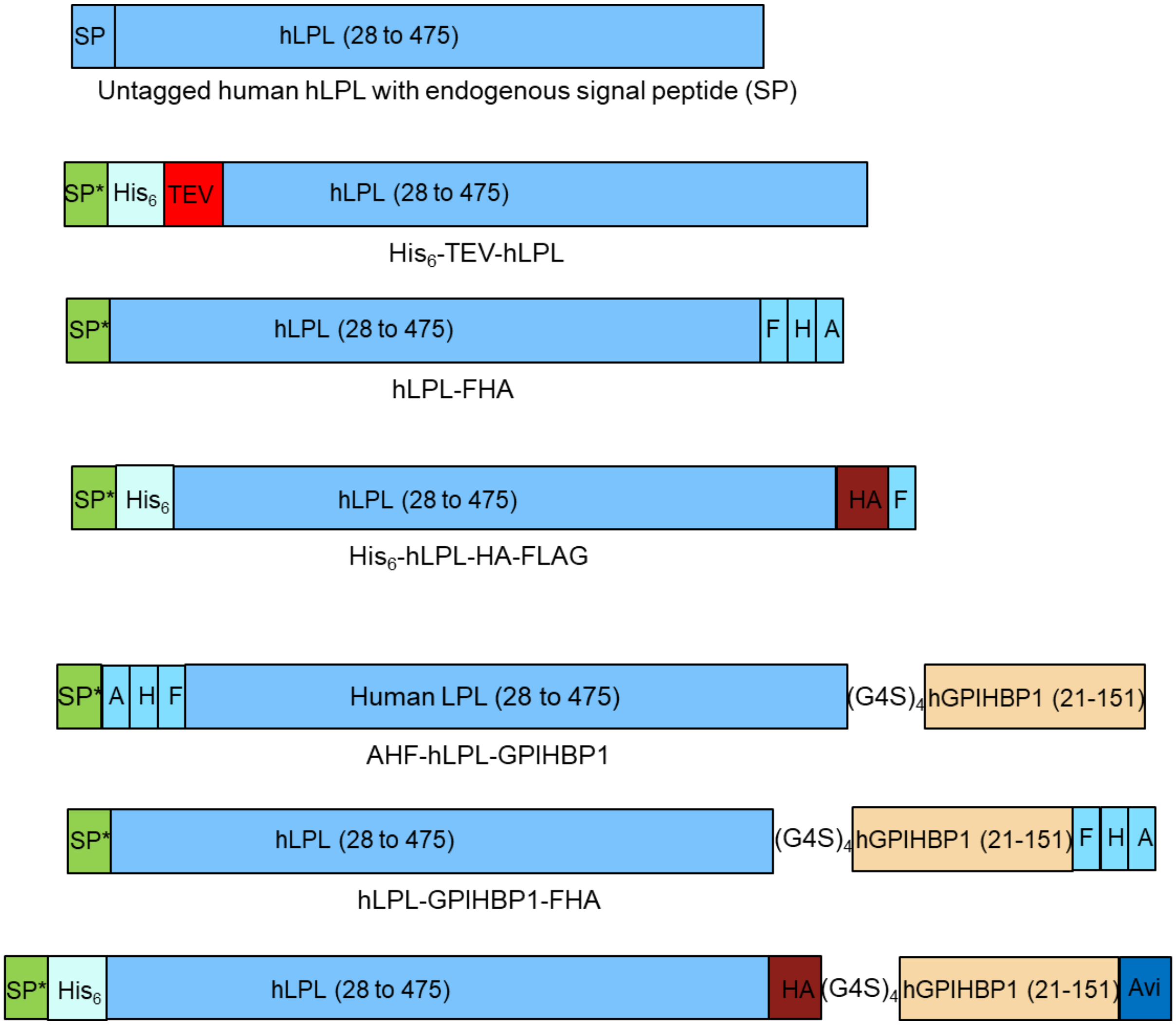

Supplementary Figure S1. Schematic representation of LPL constructs that were used in this study. SP* = mouse IgK signal peptide AHF = Avi-His_6-FLAG, FHA = FLAG-His_6-Avi, HA = hemagglutinin.

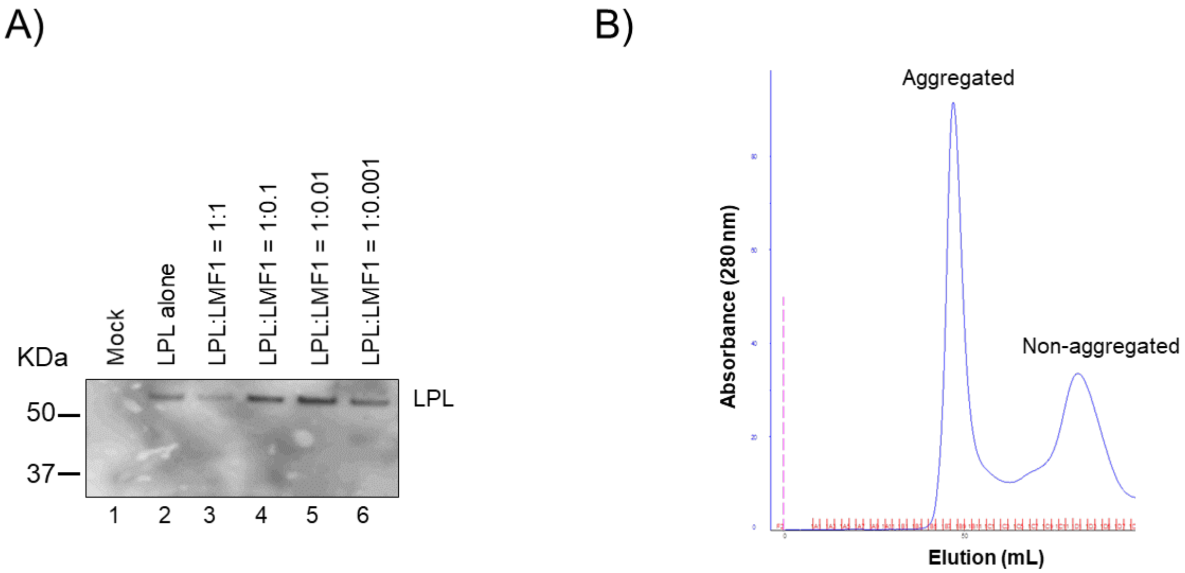

Supplementary Figure S2. (A) LMF1 alone does not substantially improve LPL expression. The image is an anti-LPL (Clone 265, Millipore) western blot of the cell culture media that compares LPL secretion by suspension-adapted HEK293T in the absence (lane 2) or in the presence of at varying amounts of LMF1 (lanes 3 to 6). (B) LPL co-expressed with LMF1 is highly aggregated. HEK293 cells were co-transfected with his-tagged LPL and LMF1 and purified using Ni-affinity, Heparin and size exclusion chromatography. During expression, dextran sulfate at concentration of 50 mg/ml was included in the media to prevent trapping of secreted protein on cell surface. The figure shows resolution of LPL using S200 gel filtration.

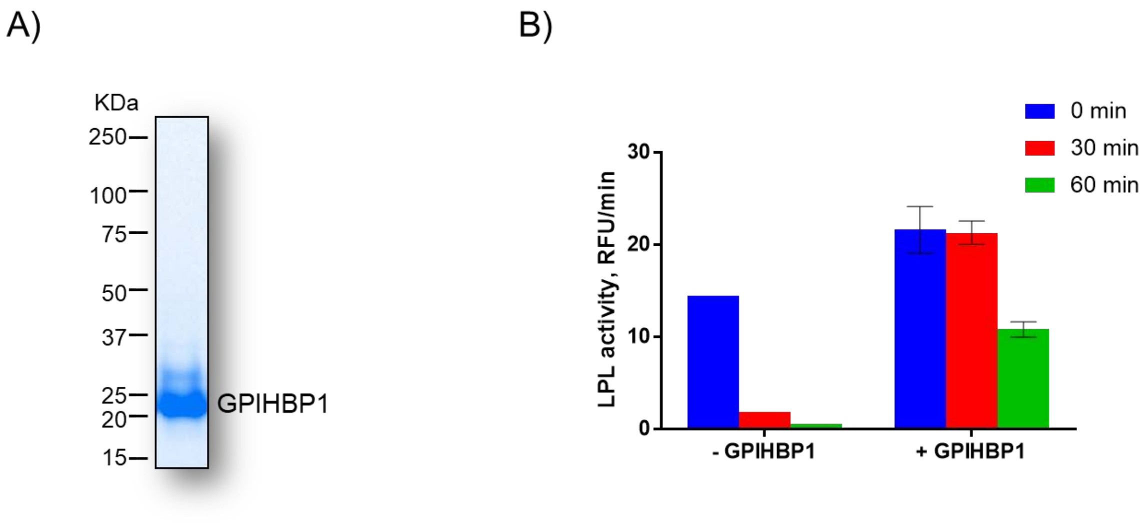

Supplementary Figure S3. (A) SDS-PAGE image showing purified GPIHBP1 with C-terminal FHA tag. HEK293 cells were co-transfected with GPIHBP1-FHA and the protein was purified using Ni-affinity chromatography. (B) GPIHBP1 stabilizes LPL. Graph comparing activity of LPL (10 nM) maintained in PBS at room temperature in the absence and presence of GPIHBP1 (12 nM). The rate of hydrolysis of resorufin butyrate (9 □M) was measured at indicated incubation times.

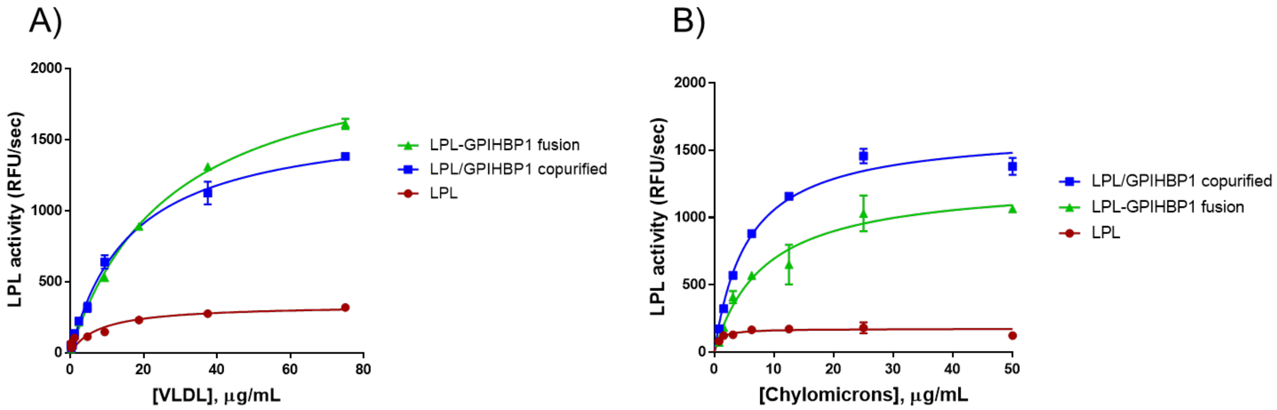

Supplementary Figure S4. LPL-GPIHBP1 fusion effectively breaks down TG in VLDL and CM. Hydrolysis of VLDL (A) and CM (B) by equimolar LPL (red), LPL/GPIHBP1 fusion (green), and LPL-GPIHBP1 complex (blue) monitored as a function of substrate concentration. The data were fit to the Michaelis-Menten equation using GraphPad Prism.

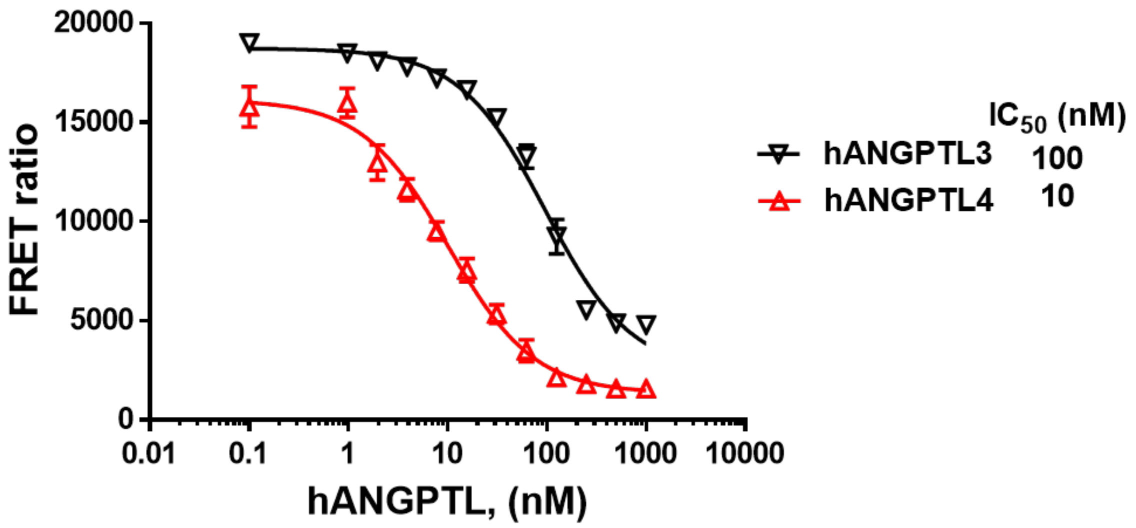

Supplementary Figure S5. ANGPTL3 and ANGPTL4 dissociate LPL/GPIHBP1 complex. LPL(HA)-GPIHBP1(Avi) complex (10 nM) was challenged with increasing concentrations of ANGPTL3 (black trace) or ANGPTL4 (red trace) proteins. The TR FRET signal was generated by using anti-HA-Tb labels and streptavidin-D2 and for LPL and GPIHBP1 respectively. The data were fit to log(antagonist) vs. response - Variable slope (four parameters) equation using GraphPad Prism.

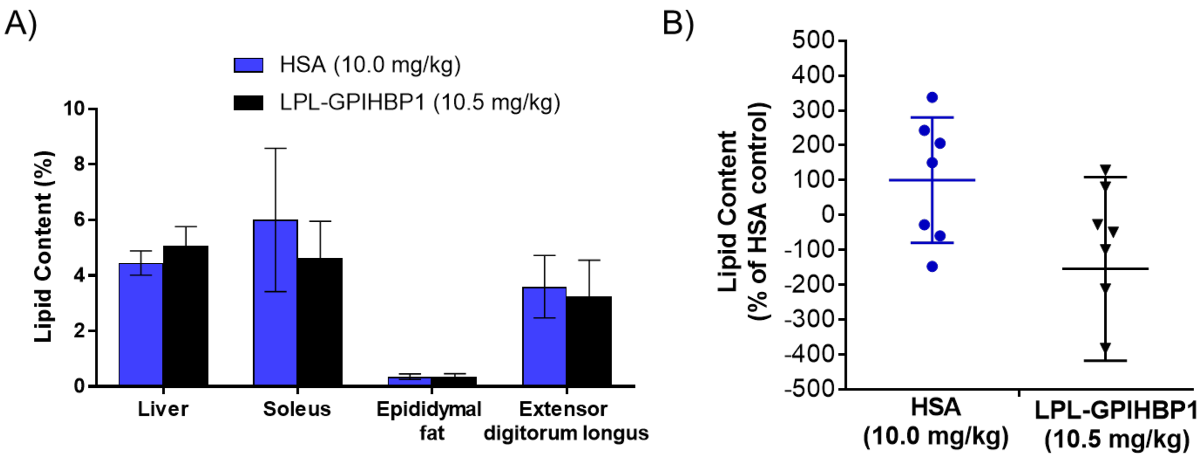

Supplementary Figure S6. Repeat LPL-GPIHBP1 SC dosing does not change tissue lipid content. (A) Lipid content in liver, soleus, epidydymal fat, and extensor digitorum longus are reported after dosing DBA/2 mice daily for 5 days with equimolar amounts of HSA (blue) or LPL-GPIHBP1 fusion protein (black). (B) Lipid content in heart. The values on Y-axes are reported relative to HSA. Differences between HSA-treated and LPL-GPIHBP1 treated groups were statistically insignificant.

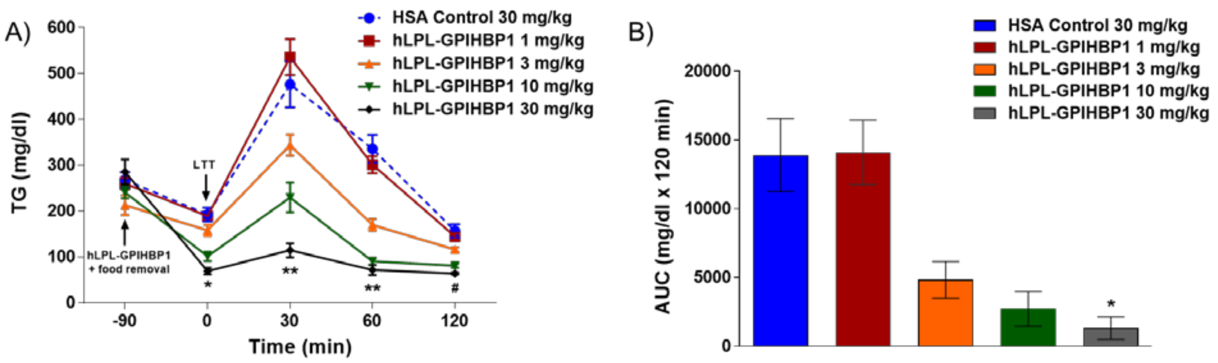

Supplementary Figure S7. LPL-GPIHBP1 fusion protein lowers triglycerides in DBA/2 mice during lipid challenge. (A) LPL-GPIHBP1 fusion protein (dosed SC, upward facing arrow) mediated lowering of TG after bolus of IV Intralipid injection (LTT, downward facing arrow). TG levels after dosing of HSA (30 mg/kg) and fusion protein (1, 3, 10 and 30 mg/kg) are plotted as a function of time. (B) Graph showing 90% AUC reduction at the highest dose of the fusion protein.
